# “Jingle All the Way!”: Sharp Foreign Bodies Embedded Within the Esophageal Mucosa During the Holiday Season

**DOI:** 10.7759/cureus.24493

**Published:** 2022-04-26

**Authors:** Lea Oliveros, Colleen McIntosh, Alex Wilsey, Sara Karjoo, Michael Wilsey

**Affiliations:** 1 Gastroenterology, Johns Hopkins All Children's Hospital, St. Petersburg, USA; 2 Pediatrics, University of South Florida (USF) Health, Tampa, USA; 3 Pediatrics, University of South Florida College of Medicine, Tampa, USA

**Keywords:** ornament, jingle bell, pediatric foreign body, flexible endoscopy, esophageal mucosa, sharp object, foreign body, holiday

## Abstract

Sharp pointed objects in the esophagus are extremely hazardous and can lead to complications such as mucosal ulcerations, perforations, obstruction, abscess, and fistula formation. Patients exhibit symptomatology based on the location within the proximal or distal esophagus. Ingestion of a sharp foreign object warrants emergent endoscopic removal, particularly when lodged in the esophagus. We present two young children, a 30-month-old male and a 10-month-old male, who underwent emergent endoscopic evaluation following the ingestion of a jingle bell and a Christmas ornament hanger, respectively. Types of ingested sharp foreign bodies may vary during the holiday season and present unique diagnostic and therapeutic challenges for pediatric physicians. Additionally, foreign body ingestions are not limited to children including teenagers and should also be considered in infants. Here, we report two young patients who ingested unique holiday ornaments and describe the management of these impacted esophageal foreign bodies.

## Introduction

Foreign bodies (FB) caught in the esophagus can lead to mucosal ulcerations, infections, or other severe complications [1]. Most foreign bodies consist of coins, jewelry, batteries, and toys [2]. Foreign body ingestion is typically diagnosed in young patients, with most studies reporting a mean age of three years old with a slight male predominance [1-4]. Presenting symptoms are variable; some of the common symptoms may include drooling, emesis, dysphagia, chest, neck or throat pain, and cough but are dependent on the size, location, and time passed since the incident [1]. Approximately, 86% of ingested foreign bodies requiring removal are located in the esophagus, 76% of which are lodged in the thoracic inlet within the upper esophagus [3]. Guidelines for different types of objects, including sharp objects, dictate whether emergent removal or close follow-up is warranted [2]. We present two cases of unique holiday foreign bodies embedded in the esophagus and discuss their management.

## Case presentation

Case 1

**Figure 1:**
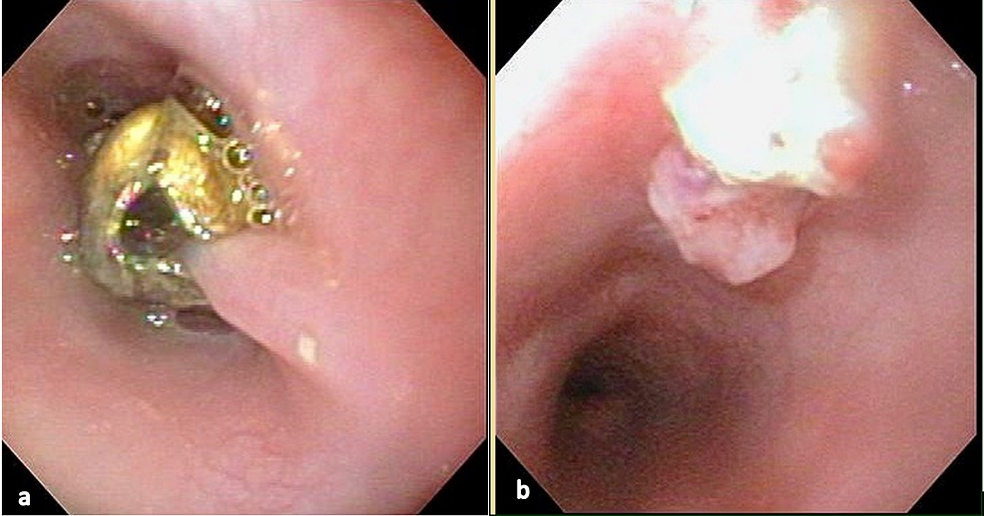
Proximal esophagus of the 30-month-old patient in Case 1 (a) Jingle bell embedded in the proximal esophagus. (b) Proximal esophagus after the removal of the jingle bell.

A 30-month-old male presented to the emergency department with a three-day history of cough, refusal of solid food, and sternal pain. Physical examination revealed the patient was febrile and noted to have significant drooling. A chest x-ray revealed a radiopaque foreign body in the esophagus that did not advance on repeat image the following morning. Endoscopy with a GIF-160 scope revealed a jingle bell embedded within the esophageal mucosa (Figure 1, Panel a). Repeated manipulation with the reusable rat-tooth, alligator-jaw, and rotatable grasping forceps (Olympus® FG-44NR-1) was required to dislodge the jingle bell from the esophageal mucosa. However, minimal tearing was observed upon re-examination (Figure 1, Panel b). Follow-up esophagram with contrast showed no perforation. The patient was discharged home the same day after tolerating oral intake.

Case 2

**Figure 2:**
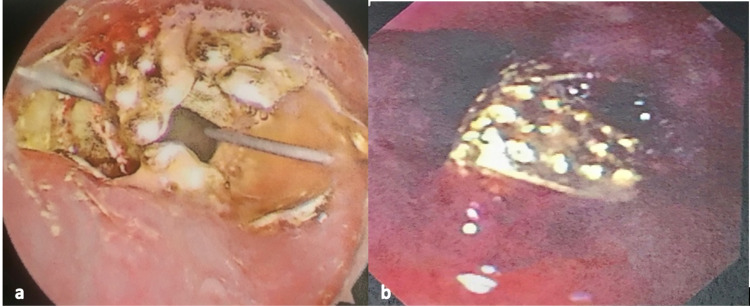
Christmas ornament embedded in the proximal esophagus of a 10-month-old patient in Case 2 (a) Christmas ornament in the proximal esophagus: cephalad view. (b) Christmas ornament embedded in the proximal esophagus following failed attempt removal with a rigid endoscope.

A 10-month-old infant male presented after witnessing choking in his playpen followed by significant drooling. A chest x-ray in the emergency department (ED) revealed a radiopaque foreign body at the level of the thoracic inlet. He was taken immediately to the operating room and intubated for airway protection. He first underwent a rigid bronchoscopy by the pediatric otolaryngology service, which identified a metal Christmas ornament hanger 1 cm distal to the upper esophageal sphincter. Unfortunately, the metal prongs of the ornament became fixed within the esophageal mucosa during the attempted removal of the object with a rigid endoscope (Figure 2). Gastroenterology service was emergently consulted for intraoperative assistance. Using a GIF-160 flexible endoscope, the object was successfully dislodged with manipulation using the reusable rat-tooth, alligator-jaw, rotatable grasping forceps (Olympus® FG-44NR-1) into the mid-esophagus. The metal hanger was then re-oriented allowing the apex of the spring to be grasped and removed in a retrograde fashion. The patient remained intubated post-procedure, given significant mucosal swelling of the site. He was extubated successfully within 12 hours. Follow-up contrast esophagram revealed no evidence of perforation. He was discharged home later that evening.

## Discussion

Foreign bodies are commonly ingested by children under five years of age [1-3]. Symptomatic ingestions allow for a prompt diagnosis if the patient lacks a significant history or witness account. The literature suggests using plain radiographs that are inexpensive and accessible [1-4]. Complication rates tend to be higher in asymptomatic patients with a delayed diagnosis over 48 hours. Children with a history of dysphagia and drooling are prone to high suspicion of FB ingestion, and radiographic evaluation should be conducted [2,4,5]. Radiopaque inorganic objects are better identified on x-rays. If the object is radiolucent, it can be located using an esophagram with contrast material; however, this modality may delay treatment significantly and affect visualization during endoscopy [1,2].

Impacted foreign bodies in the esophagus may cause complications as far as 18%, with esophageal perforation rates varying from 2% to 15% [6]. Complications of FBI include mucosal ulcerations, perforations, obstruction, abscess, and fistula formation. Removal of foreign bodies using flexible upper endoscopy has a high rate of treatment success in pediatric patients [6]. Therefore, the employment of emergent endoscopic removal using flexible endoscopy is warranted following the ingestion of sharp foreign bodies. Additionally, foreign bodies causing respiratory distress or impact for at least 24 hours require emergent removal. Follow-up esophagram contrast studies allow confirmation of an intact mucosa [2].

The type and frequency of foreign bodies ingested by children are often dependent upon seasonal and cultural factors [2]. Unlike most sharp object ingestions in the United States, which include safety pins, straight pins, and nail ingestions [1-3], our patients ingested unique holiday ornaments. Although prior reports suggested children at this age are prone to swallow holiday foreign bodies [7,8], being embedded within the esophageal mucosa is a rare occurrence. Patients are more likely to be symptomatic when a foreign body is lodged in the mid or upper esophagus, often presenting with symptoms of pain, dysphagia, and drooling [1-3]. However, up to 50% may remain asymptomatic for several weeks after FB ingestion [2]. Symptomatology in our patients led to prompt evaluation with imaging and emergent endoscopic retrieval as per the current North American Society for Pediatric. Gastroenterology, Hepatology, and Nutrition (NASPGHAN) guidelines [2].

## Conclusions

Types of ingested sharp foreign bodies may vary during the holiday season and present unique diagnostic and therapeutic challenges for pediatric providers. Additionally, foreign body ingestions are not limited to toddlers and should also be considered in infants. Here, we report two young patients who ingested sharp holiday ornaments that led to esophageal impaction and embedding into the esophageal mucosa. Further reporting of endoscopic removal for these impacted holiday foreign bodies adds to the literature to help evaluate the effectiveness of current endoscopic treatment strategies and may help guide future treatment guidelines.
